# Napabucasin Reduces Cancer Stem Cell Characteristics in Hepatocellular Carcinoma

**DOI:** 10.3389/fphar.2020.597520

**Published:** 2020-12-03

**Authors:** Ya Li, Qiuju Han, Huajun Zhao, Quanjuan Guo, Jian Zhang

**Affiliations:** Institute of Immunopharmaceutical Sciences, School of Pharmaceutical Sciences, Shandong University, Jinan, China

**Keywords:** napabucasin, stat3, hepatocellular carcinoma, cancer stem cells, stemness, hepatitis B virus

## Abstract

Hepatocellular carcinoma (HCC) is the most common type of primary liver cancer. *Cancer* stem cells (CSCs) are a rare population with self-renewal and multipotent differentiation capacity, and reside among the more differentiated cancer cells. CSCs are associated with tumor recurrence, drug resistance and poor prognosis. The aim of this study was to determine the efficacy of napabucasin against HCC and elucidate the underlying molecular mechanisms. Napabucasin significantly decreased the viability of HCC cells *in vitro* by inducing apoptosis and cell cycle arrest. In addition, it suppressed CSC-related gene expression and spheroid formation *in vitro*, indicating depletion of CSCs. The anti-neoplastic effects of napabucasin was also evident in homograft tumor-bearing mouse models. Our findings provide the scientific basis of conducting clinical trials on napabucasin as a new therapeutic agent against HCC.

## 1 Introduction

Hepatocellular carcinoma (HCC) is the most common type of primary liver cancer, and chronic liver diseases and cirrhosis are its underlying pathologies. The annual mortality rate of liver cancer is 700,000 worldwide, and the number of newly diagnosed HCC is estimated to increase further in the coming decades (Balogh et al., 2016; Rawla et al., 2018; Asrani et al., 2019). Since it is asymptomatic in the early stages, HCC is usually diagnosed in its advanced stage, which precludes the possibility of surgical resection (Daher et al., 2018). At present, sorafenib is the only chemotherapeutic agent approved for treating advanced HCC, although it provides a survival benefit of only a few months (Geschwind and Chapiro, 2016; Samonakis and Kouroumalis, 2017; Daher et al., 2018). Therefore, early diagnosis and treatment of HCC is currently a major research focus.

*Cancer* stem cells (CSCs) are a rare subpopulation of tumor cells that have the capacity to self-renew and differentiate into multiple lineages. They exist along with the larger differentiated cancer cells, and are associated with tumor recurrence and poor prognosis (Jiang et al., 2012; Bao et al., 2013). The self-renewal of CSCs is regulated by transcription factors like Nanog, SOX2, Oct4 and Klf-4 (Frank et al., 2010). CSCs are also responsible for tumor radio-resistance, chemoresistance and invasion (Phi et al., 2018). Therefore, preventing liver CSCs formation is a viable therapeutic strategy against HCC.

Signal transducer and activator of transcription 3 (STAT3) is a latent cytoplasmic transcription factor that translocates to the nucleus following tyrosine phosphorylation at position 705 and dimerization (Karras et al., 1997). STAT3 activation is tightly controlled in healthy cells, and is constitutively activated during tumorigenesis, where in it upregulates genes involved in tumor cell proliferation, invasion, migration, and angiogenesis (Kamran et al., 2013). STAT3 is overexpressed in approximately 60% of the HCC tissues, and is associated with poor prognosis (Kim et al., 2004). Consistent with this, blocking the STAT3 signaling pathway in human HCC cells using a decoy oligonucleotide induced apoptosis (Sun et al., 2008). Furthermore, STAT3 controls the expression of the CSC transcription factors like October 4 and Nanog (Yin et al., 2015). Won et al. reported that interleukin-6 (IL-6)-mediated STAT3 signaling and hypoxia upregulated CD133 in HCC cells and promoted tumor progression (Won et al., 2015). IL-6/STAT3-dependent Oct4 expression has also been observed in HCC cell lines and tumor homografts (Lai et al., 2018). Therefore, STAT3 is a promising therapeutic target in HCC.

Napabucasin is a neoteric small molecule that targets the STAT3 pathway, and has shown encouraging results in phase III clinical trials on metastatic colorectal carcinoma, pancreatic cancer, gastric cancer and non-small cell lung cancer (Sonbol et al., 2019). In addition, napabucasin prevented relapse and metastasis of human tumor xenografts by inhibiting CSCs (Li et al., 2015; Beyreis et al., 2019). However, the efficacy of napabucasin in HCC and the underlying molecular mechanisms have not been elucidated. Here, we found that napabucasin suppressed HCC cell growth *in vitro* and *in vivo*, and was highly toxic to HCC-derived CSCs. Our findings indicate that napabucasin is a promising agent for treating HCC.

## 2 Materials and Methods

### 2.1 Cell Lines and Culture

The human HCC cell lines Huh7, HepG2 and the murine Hepa1-6 cells were purchased from the Cell Bank of Type Culture Collection of the Chinese Academy of Sciences (Shanghai, China), human HCC cell line HepG2.2.15 was kindly provided by professor Chun-Hong Ma (School of Basic Medical Science, Shandong University, China). All of the cells were cultured in DMEM or RPMI-1640 medium supplemented with 10% fetal bovine serum (FBS; Biological Industries, CT, USA) and 1% penicillin and streptomycin (Solarbio, Beijing, China) at 37°C under 5% CO_2_. The HepG2.2.15 cells were selectively cultured with 200 mg/ml antibiotic G418 (Sigma-Aldrich, St. Louis, MS, USA).

### 2.2 Drugs

The STAT3 inhibitor napabucasin, cryptotanshinone and oxaliplatin were purchased from Selleck (Selleckchem, Houston, TX, USA). Napabucasin and cryptotanshinone were dissolved in dimethyl sulfoxide (DMSO) (Solarbio) at the final concentration of 50 mM as stock solution and stored at –20°C. Oxaliplatin was dissolved in saline at 10 mM for the stock solution.

### 2.3 Cell Viability Assay

HCC cells were seeded in a 96-well plate at the density of 1 × 10^4^ cells/200 µL/well in DMEM or RPMI-1640 medium containing different concentrations of napabucasin, cryptotanshinone or oxaliplatin. After culturing the cells for 24, 48 or 72 h, cell counting kit-8 (CCK8, Beyotime Biotechnology, Shanghai, China) was added to each well and the cells were incubated for another 1 h. Absorbance was measured at 450 nm using a Synergy^™^ two Multi-Mode microplate spectrophotometer (BioTek, Winooski, VT, USA).

### 2.4 Apoptosis Assay

HCC cells were seeded in a 6-well plate at the density of 2 × 10^5^ cells per well, cultured overnight, and incubated with napabucasin for an additional 4 or 12 h. The cells were harvested, washed with phosphate buffered saline (PBS), and stained using the Annexin V-FITC Apoptosis Detection Kit (Sungene biotech, Tianjin, China) according to the manufacturer’s instructions. The stained cells were acquired by FACSCalibur flow cytometer (BD Biosciences, Franklin Lakes, NJ, USA), and the percentages of apoptotic cells were analyzed using the FlowJo software (Tree Star, San Carlos, CA, USA).

### 2.5 Cell Cycle Analysis

HCC cells were harvested and fixed with 70% ethanol in ddH_2_O at room temperature for 2 h, washed with PBS, and incubated with propidium iodide (PI) solution (Sungene biotech, Tianjin, China) for 30 min. The cells were sorted by FACSCalibur flow cytometer (BD Biosciences), and the cell cycle distribution was analyzed using ModFit LT 5.0.

### 2.6 Hoechst Staining

Suitably-treated HCC cells were fixed with 4% paraformaldehyde (Sinopharm Chemical Reagent limited corporation, Shanghai, China) and stained with 1 μg/ml Hoechst 33342 (Beyotime Biotechnology). The cells were observed by fluorescence microscopy (Olympus Corporation, Tokyo, Japan).

### 2.7 Colony Formation Assay

HCC cells were seeded in a 6-well plate at the density of 500 cells/well, and incubated with DMSO or varying concentrations of napabucasin for 8 h, followed by a 7-days culture. The cells were fixed with 4% paraformaldehyde (Sinopharm Chemical Reagent limited corporation) and stained with 1% crystal violet solution (Solarbio) for 10 min, and the colonies (>100 cells) were counted under a microscope (Olympus Corporation).

### 2.8 Spheroid Culture

For testing self-renewal capacity, a total of 100 single HCC cells were seeded in each well of ultra-low attachment 96-well plate (Corning Incorporated, Corning, NY, USA) in complete suspension culture medium, DMEM/F12 (Invitrogen, Carlsbad, CA, USA) containing 2% B27 (Invitrogen), 1% N2 (Invitrogen), 20 ng/ml epidermal growth factor (EGF; Pep-Tech Corporation, Burlington, MA, USA), and 20 ng/ml basic fibroblast growth factor (bFGF; Pep-Tech Corporation) with DMSO or various concentrations of napabucasin. The multicellular spheroids larger than 80 μm were counted after 7 days.

For extreme limiting dilution analysis (ELDA), the HCC multicellular spheroids were dissociated to single cells and seeded at different densities (1, 10, 50 and 100/well) in complete suspension culture medium with DMSO or 1 µM napabucasin in ultra-low attachment 96-well plates. There were nine wells per cell density and cultured for 7 days. The numbers of wells with at least one multicellular spheroids (diameter > 80 μm) were counted in a blinded manner. Frequencies of sphere-initiating cells were calculated by ELDA online program (http://bioinf.wehi.edu.au/software/elda/) (Hu and Smyth, 2009).

To evaluate the effect of napabucasin on HCC multicellular spheroids, a total of 500 single HCC cells were seeded in each well of ultra-low attachment 6-well plate (Corning Incorporated) in complete suspension culture medium. The multicellular spheroids larger than 80 μm were harvested for analysis 7 days after growth. For subsequent treatment with napabucasin, HCC multicellular spheroids (10 per well) were seeded into ultra-low attachment 96-well plate (Corning Incorporated) in complete suspension culture medium with DMSO or various concentrations of napabucasin. The images were captured with the JuLI^TM^ Stage Real-Time Cell History Recorder (NanoEnTek, Seoul, South Korea).

### 2.9 Western Blotting

Cellular proteins were extracted using RIPA buffer (Beyotime Biotechnology), and equal amounts per sample were separated by SDS-PAGE in an 8% gel. The protein bands were transferred to a polyvinylidene-fluoride (PVDF) membrane (Millipore, Burlington, MA, USA) that was blocked with 5% fat-free milk in PBS, and then incubated overnight with anti-p-STAT3 (Tyr705) (Cell Signaling Technology, Danvers, MA, USA), anti-STAT3 (Cell Signaling Technology) and anti-GAPDH (Beyotime Biotechnology) antibodies (diluted 1:1000). The blots were further incubated with HRP-conjugated goat anti-rabbit or mouse IgG secondary antibodies (Beyotime Biotechnology) and developed with enhanced chemiluminescence reagent (Millipore, Billerica, MA, USA). The density of the bands was calculated using the Image-Lab software (Version 3.0, Bio-Rad).

### 2.10 Quantitative Real-Time PCR (qRT-PCR)

Suitably-treated HCC cells were washed with 1 × PBS 3 times before collected. Total RNA from HCC cells was extracted using TRIzol reagent (Invitrogen) and reverse transcribed to cDNAs with the SuperScript™ IV First-Strand Synthesis System (Invitrogen, Carlsbad, CA, USA). Quantitative RT-PCR was performed using SYBR Green I Master (Roche, Basel, Switzerland) on a LightCycler^®^ 480 System according to the manufacturer’s instructions. The relative mRNA levels were calculated using the 2^−ΔΔCt^ method. Primer sequences are shown in Supplementary Table 1.

### 2.11 Mice and Tumor Models

C57BL/6J male mice (6-week-old) were purchased from Huafukang Laboratories (Beijing, China), and all animal experiments were performed according to the recommendations of the Ethical Committee of Shandong University. The mice were each subcutaneously injected in their left axilla with 5 × 10^6^ Hepa1-6 cells, and the tumors were measured every 2 days. The tumor volume (mm^3^) was calculated as length × width^2^/2. The tumor-bearing mice were intraperitoneally injected with 20 mg/kg napabucasin every 2 days in a 1:8:1:10 napabucasin:poly (ethylene glycol)-300:Tween 80:saline mix, or equal volume of the vehicle. The mice were euthanized after eight injections, and the tumors were harvested for further analysis.

### 2.12 Isolation of Mouse Tumor Cells

HCC tumor tissues were harvested, minced, and digested with 0.1 mg/ml DNAse (Roche), 1 mg/ml type IV collagenase (Invitrogen) and 0.5% hyaluronic acid (Solarbio) for 1 h at 37 °C. The homogenates were passed through 200-mesh stainless steel strainers to obtain single cell suspension. The tumor cells were pelleted with 700 rpm for 1 min at the bottom of the centrifuge tube. For qRT-PCR analysis, total RNAs were extracted with TRIzol. For flow cytometry analysis, cells were fixed with 4% paraformaldehyde and 0.5% TritonX-100 (Sigma), blocked with 0.1% rat serum in PBS and stained with fluorescence-conjugated anti-Ki67 antibody (Clone 16A8, Biolegend, San Diego, CA, USA) for 40 min at 4°C. The stained cells were acquired using a FACSCalibur flow cytometer (BD Biosciences) and analyzed by FlowJo software (Tree Star).

### 2.13 Histology and Immunohistochemistry

Tumor homografts were excised after treatment, fixed with 4% paraformaldehyde, embedded in paraffin, and cut into 4-μm thick sections. Hematoxylin and eosin (H&E) staining was performed as per standard protocols, and the necrotic areas were observed under a light microscope (Olympus Corporation). For TUNEL staining, the tissue slices were deparaffinized with xylene, successively rehydrated in 100%, 90%, 80% and 70% ethanol, and incubated with 20 μg/ml Proteinase K without DNase for 30 min. After washing with PBS, the slices were incubated with the TUNEL working solution for 1 h at room temperature, and observed under a fluorescence microscope (Olympus Corporation).

### 2.14 STAT3 Interference

The sequence 5’-TGC​TGA​CCA​ACA​ATC​CCA​A-3’ (residues 1663–1681 in the human STAT3 cDNA sequence, GenBank accession no. NM_003150) as a target to construct shRNA for stable knockdown of STAT3 was cloned into pLKO.1 plasmid. The recombinant lentiviruses expressing the STAT3 interference fragment were produced in 293T cells and used to infect human HCC cells in the presence of 8 μg/ml of polybrene (Sigma-Aldrich). Then HCC cells were collected for further analysis after culturing for 48 h.

### 2.15 Statistical Analysis

Different groups were compared with Student’s t-test or analysis of variance (ANOVA) as appropriate. The half maximal inhibitory concentration (IC_50_) values were calculated by PASW Statistics 18.0.0. All data are expressed as mean ± standard deviation (SD), and considered statistically significant at *p* < 0.05.

## 3 Results

### 3.1 Napabucasin Inhibits HCC Cell Growth *in vitro*

**FIGURE 1:**
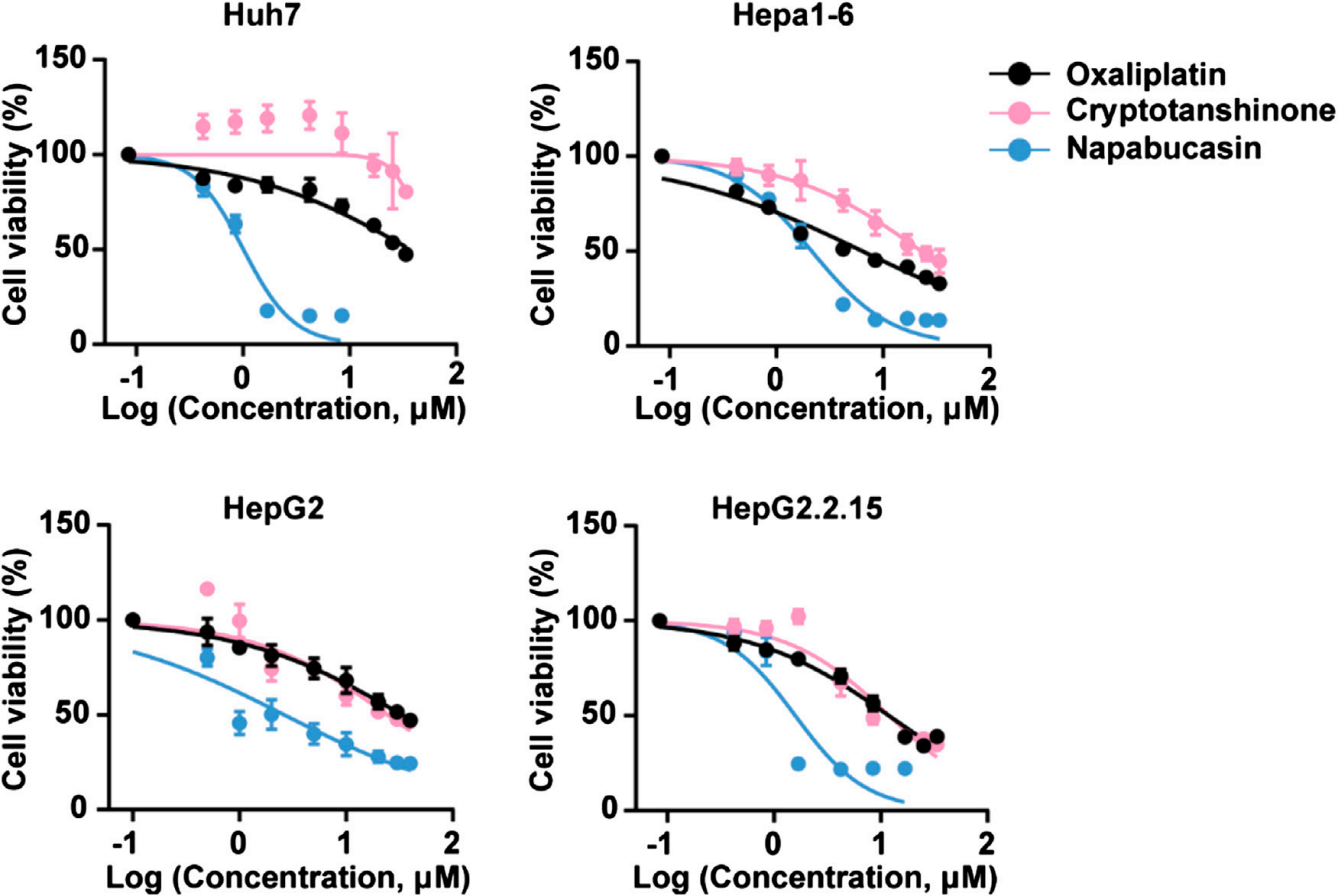
Napabucasin decreases the viability of HCC cells *in vitro*. Viability of HCC cells incubated with DMSO or indicated concentrations of oxaliplatin, cryptotanshinone and napabucasin for 48 h. Data are representative of three independent experiments.

**FIGURE 2:**
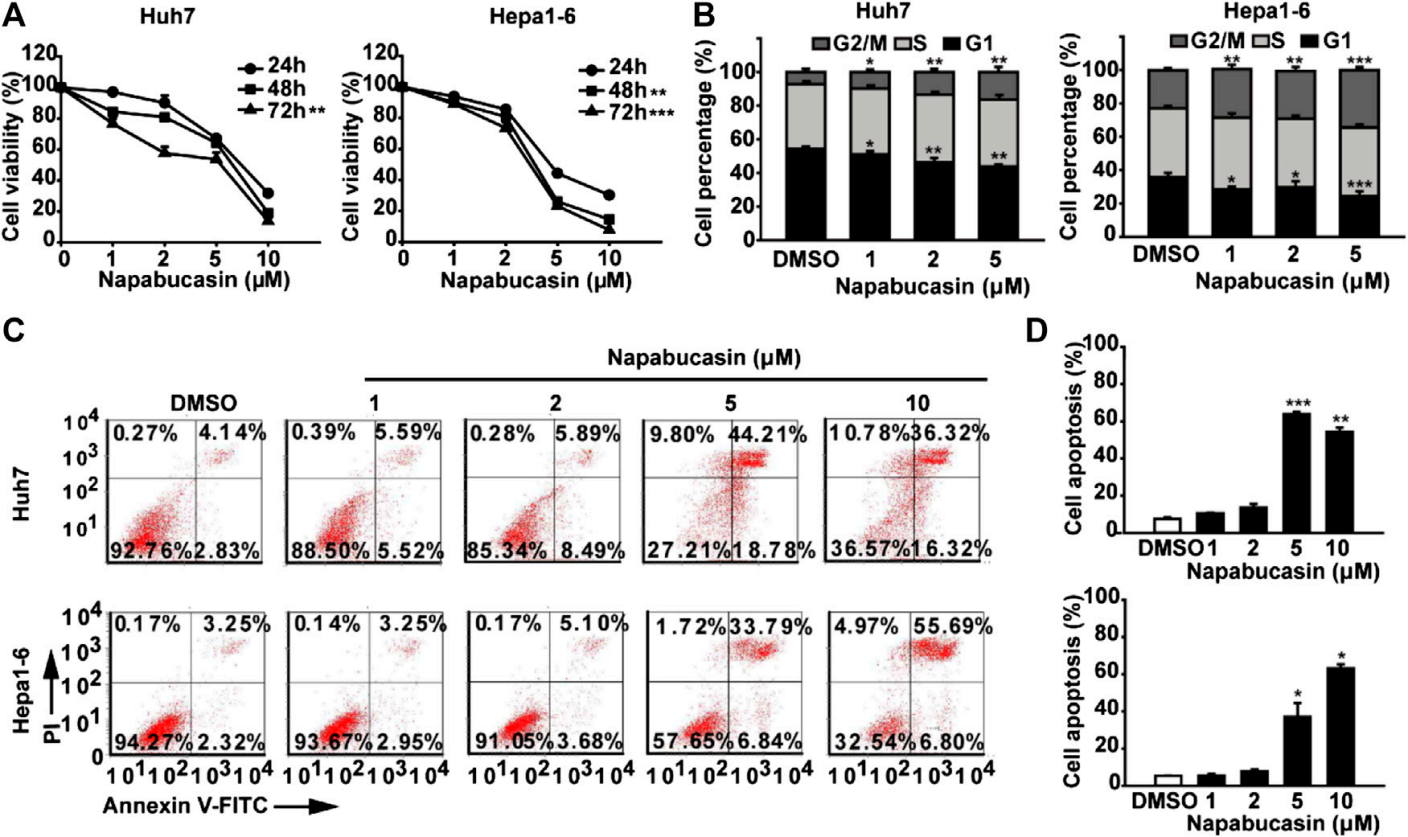
Napabucasin induces the apoptosis and cell cycle arrest of HCC cells. **(A)** Viability of Huh7 and Hepa1-6 cells treated with DMSO or the indicated concentrations of napabucasin for 24, 48, and 72 h. **(B)** Cell cycle distribution of Huh7 and Hepa1-6 cells after treatment with DMSO or napabucasin for 24 h. The percentage of the cells at different phases are shown. **(C)** Percentage of apoptotic Huh7 and Hepa1-6 cells treated with DMSO or napabucasin for 12 h. Data are shown as mean ± SD of three independent experiments (*P < 0.05, **P < 0.01 and ***P < 0.001).

The potential cytotoxic effect of napabucasin on HCC cells was evaluated in terms of the proliferation ability of multiple human and murine HCC cell lines, and compared to that of oxaliplatin and the plant-derived STAT3 inhibitor cryptotanshinone (Liu et al., 2017; Qin et al., 2017; Ji et al., 2019; Zhu et al., 2019). As shown in Figure 1, all drugs significantly decreased the viability of HCC cells in a concentration-dependent manner. However, napabucasin exhibited a stronger suppressive effect compared to either cryptotanshinone or oxaliplatin, and had a significantly lower IC_50_ value (Table1). As shown in Figure 2A, napabucasin significantly decreased the viability of Huh7 and Hepa1-6 cells in a time- and concentration-dependent manner. Consistent with this, the proportion of napabucasin-treated cells in the G1-phase was significantly lower compared to that of the untreated controls, which corresponded to an increase in the G2/M-phase cells (*P* < 0.05; Figure 2B). Furthermore, the apoptosis rates in Huh7 cells increased to 11.11%, 14.38% and 62.99% respectively within 12 h of exposure to 1, 2 and 5 µM napabucasin (Figure 2C), and similar results were obtained with Hepa1-6 cells as well. Taken together, napabucasin inhibited HCC cell growth in a concentration-dependent manner by inducing apoptosis and G2/M-phase arrest.

**TABLE 1:** IC_50_ of napabucasin, oxaliplatin and cryptotanshinone in HCC cell lines.

IC_50_ (μM)	Huh7	HepG2	HepG2.2.15	Hepa1-6
Oxaliplatin	44.09 ± 0.79	33.53 ± 0.91	14.09 ± 0.34	7.56 ± 2.7
Cryptotanshinone	61.31 ± 2.3	24.49 ± 0.97	14.19 ± 1.59	26.95 ± 1.17
Napabucasin	1.2 ± 0.13	2.64 ± 0.03	1.88 ± 0.17	2.53 ± 0.23

### 3.2 Napabucasin Decreases Colony and Spheroid Formation Abilities of HCC Cells

**FIGURE 3:**
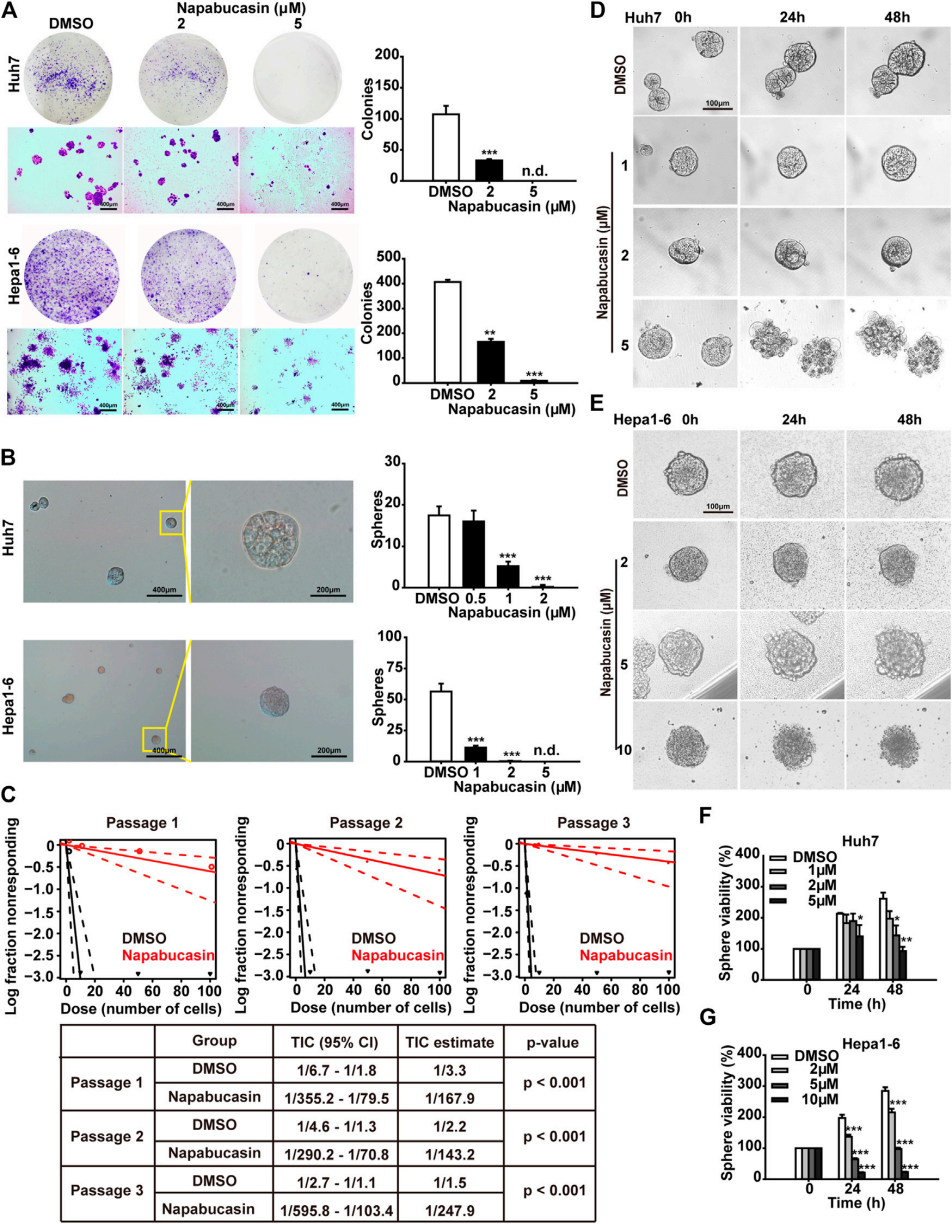
Napabucasin suppresses sphere formation and viability of HCC cells. **(A)** Representative images of crystal violet-stained colonies (>100 cells) of Huh7 and Hepa1-6 cells after treatment with DMSO or indicated concentrations of napabucasin. **(B)** Representative images of multicellular spheroids larger than 80 μm **(left)** and quantification of spheroids derived from DMSO- or napabucasin-treated Huh7 and Hepa1-6 cells **(right)**. n.d., not detected. **(C)** Limiting dilution sphere assay of Huh7 cells under DMSO or 1 µM napabucasin from passage one to three. ELDA analysis plot and relative table reporting the TIC percentage and estimation are shown. TIC: tumor-initiating cells. **(D)**, **(E)**. Representative real-time images of Huh7 and Hepa1-6 spheroids in the presence of DMSO or napabucasin. **(F)**, **(G)**. Viability of the DMSO or napabucasin-treated spheroids after 24 or 48 h. Data are shown as mean ± SD of three independent experiments (*P < 0.05, **P < 0.01 and ***P < 0.001).

To determine the potential effects of napabucasin on HCC metastasis, we assessed the colony-forming capacity of these cells following drug treatment. As shown in Figure 3A, napabuacsin significantly decreased the number and size of colonies formed by both Huh7 and Hepa1-6 cells in a concentration-dependent manner. Since metastasis is dependent on CSCs, we next analyzed the ability of the napabucasin-treated HCC cells to form spheroids in suspension. Compared to the DMSO-treated controls, napabucasin blocked spherogenesis of HCC cells (Figure 3B), and such ability is further enhanced along with the serial passages (Figure 3C), indicating that this drug can inhibit the self-renewal and proliferation of the CSC-like cells. Furthermore, the viability of the multicellular spheroids derived from Huh7 cells was significantly reduced by napabucasin, and the cell masses were obliterated at the high concentration of 5 μM (Figures 3D,F). Similar results were seen with the Hepa1-6 cells (Figures 3E,G). Taken together, napabucasin showed an inhibitory effect on HCC metastasis, and the “stemness-high” HCC cells were particularly sensitive to the drug.

### 3.3 Napabucasin Downregulates p-STAT3^Tyr705^ and Stemness Markers in HCC Cells

**FIGURE 4:**
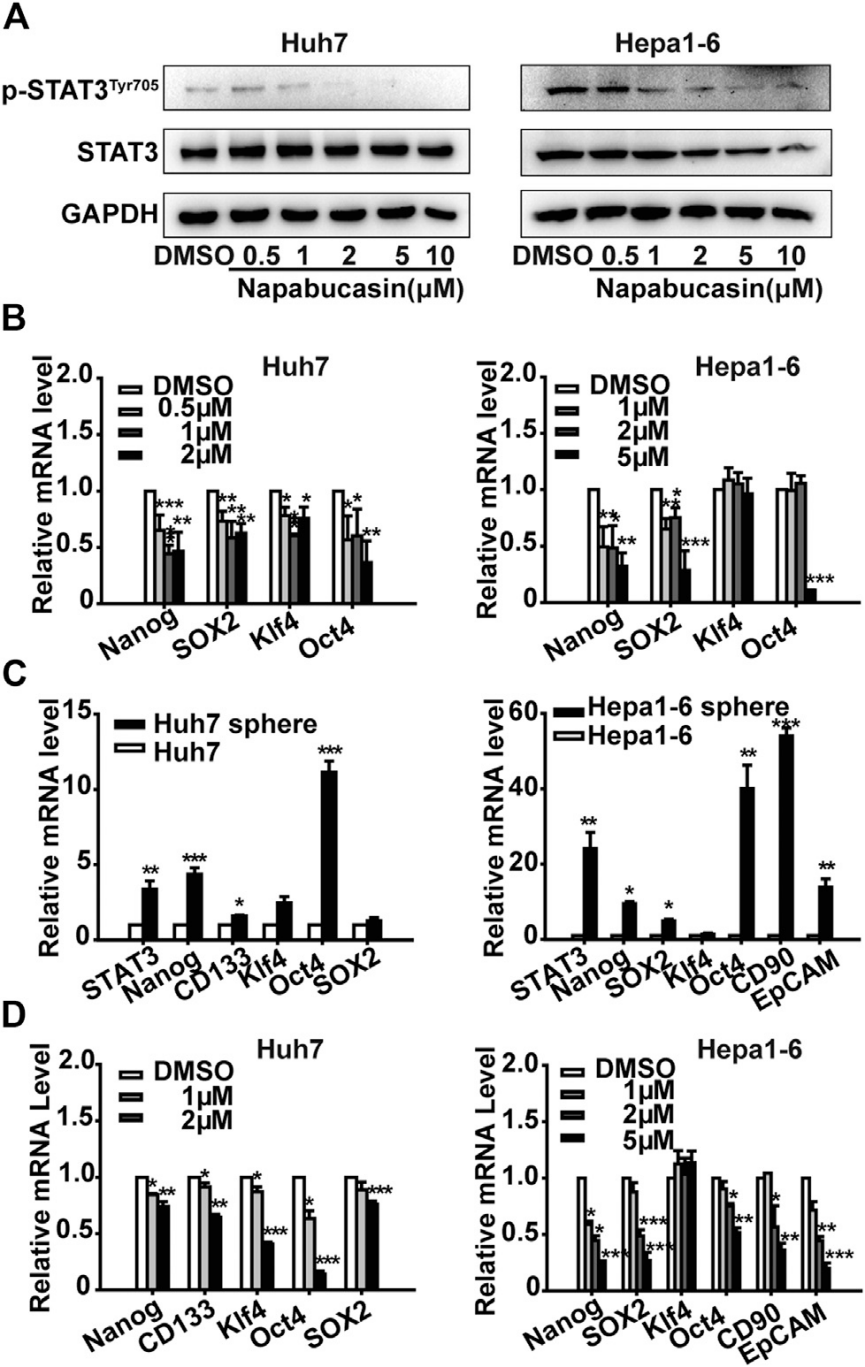
Napabucasin down-regulates p-STAT3^Tyr705^ and stemness markers. **(A)** Immunoblot showing STAT3 and p-STAT3^Tyr705^ levels in Huh7 and Hepa one to six cells treated with different concentrations of napabucasin for 12 h. **(B)** STAT3 and stemness marker mRNAs in the suitably treated Huh7 and Hepa1-6 cells. Data were normalized to β-actin. **(C)** STAT3 and stemness marker expression in spheroid derived from Huh7 and Hepa1-6 cells. **(D)** Stemness marker expression in Huh7 or Hepa1-6 cell-derived spheres treated with indicated concentrations of napabucasin for 12 h. Data are shown as mean ± SD of three independent experiments (*P < 0.05, **P < 0.01 and ***P < 0.001).

The TCGA database showed an elevated expression of stemness makers such as Nanog, SOX2, Oct4 and CD90 in HCC patients (Supplementary Figure 1). *In vitro*, the mRNA levels of Nanog, CD133, SOX2, Klf4, Oct4 and SOX2 were significantly decreased by STAT3 interference in HCC cells (Supplementary Figure 2). To further elucidate the molecular mechanisms underlying napabucasin action, we next analyzed the changes in the expression levels of STAT3 and stemness markers in the napabucasin-treated cells. Napabucasin decreased *p*-STAT3^Tyr705^ levels in Huh7 and Hepa1-6 cells in a concentration-dependent manner, but had little effect on total STAT3 expression (Figure 4A). In addition, the expression levels of Nanog, SOX2, Klf4 and Oct4 mRNA were also significantly decreased in the napabucasin-treated HCC cells in a concentration-dependent manner (Figure 4B). Furthermore, compared to the adherent cultures, STAT3, Nanog and Oct4 mRNAs were significantly upregulated in the Huh7 and Hepa1-6 cell-derived spheroids grown in suspension (Figure 4C), which was decreased by napabucasin in a concentration-dependent manner (Figure 4D). Therefore, napabucasin specifically inhibits the stemness-high HCC cells by inactivating STAT3 and downregulating multiple CSC-related transcription factors, and is expected to yield therapeutic effects in clinical.

### 3.4 Napabucasin Induces HBV^+^ HCC Cell Apoptosis and Decreases Spheroid Formation

**FIGURE 5:**
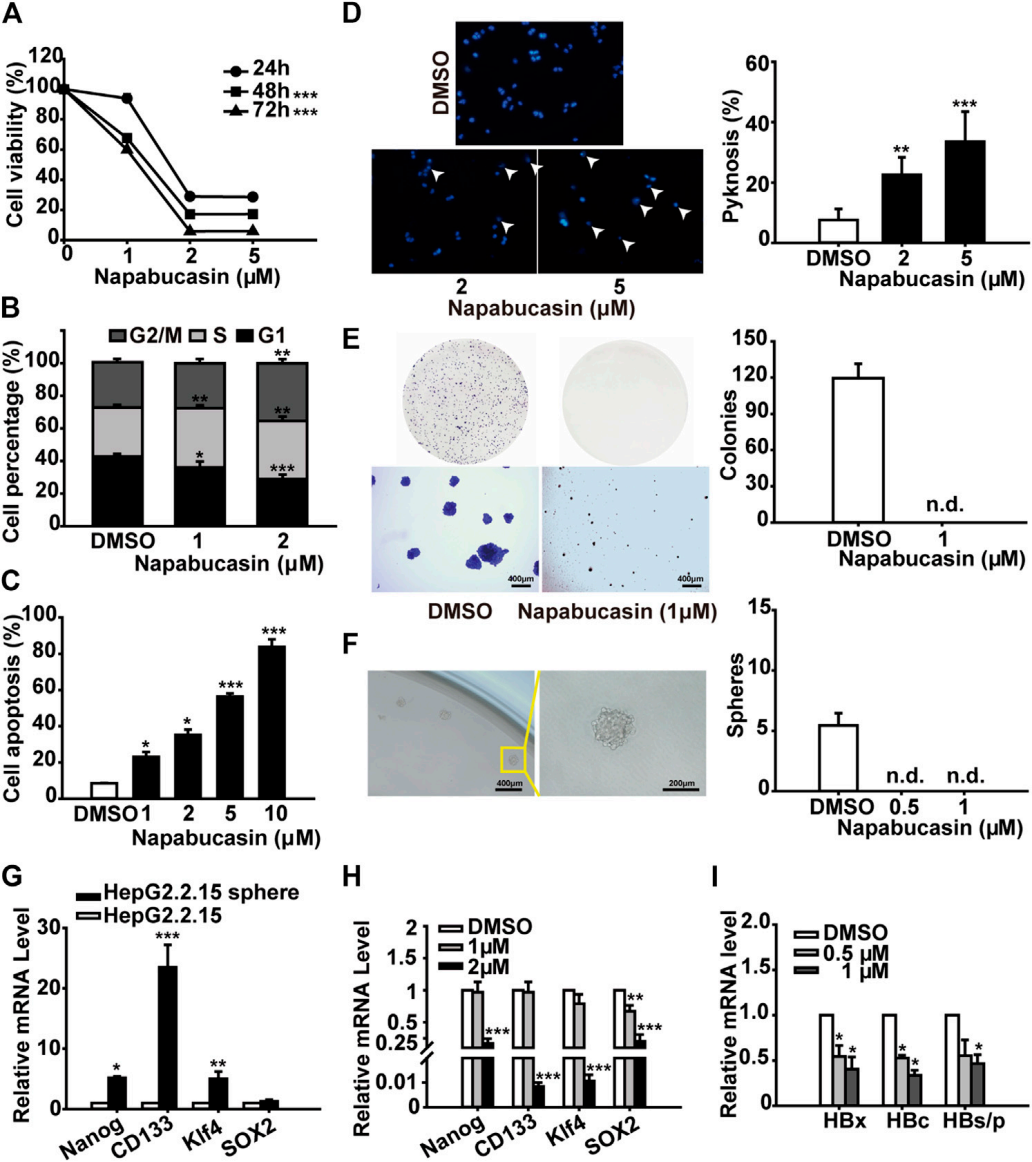
Napabucasin induces apoptosis in HBV^+^ HCC cells and decreases spheroid formation. **(A)** Viability of HepG2.215 cells treated with DMSO or indicated concentrations of napabucasin for 24, 48 and 72 h. **(B)** Cell cycle distribution of HepG2.215 cells treated with DMSO or napabucasin for 24 h. **(C)** Percentage of apoptotic HepG2.215 cells following treatment with DMSO or napabucasin for 12 h. **(D)** Representative images (×40 magnification) of Hoechst 33342-stained HepG2.215 cells treated with napabucasin for 6 h **(left)** and quantification of pyknosis **(right)**. **(E)** Representative images of colonies formed by DMSO or napabucasin-treated HepG2.2.15 cells **(left)** and quantification of colonies **(right)**. **(F)** Representative images of multicellular spheroids larger than 80 μm **(left)** and quantification of spheroids derived from DMSO- or napabucasin-treated HepG2.215 cells **(right)**. **(G)** Stemness marker expression in the spheres derived from HepG2.215 cells. **(H)** Stemness marker expression in HepG2.215 cell-derived spheres treated with indicated concentrations of napabucasin for 12 h. Data were normalized to β-actin. **(I)** HBx, HBc and HBs/p levels in HepG2.215 cells treated with napabucasin for 12 h. Data are shown as mean ± SD of three independent experiments (*P < 0.05, **P < 0.01 and ***P < 0.001). n.d., not detected.

Hepatitis B virus (HBV) infection is the most common underlying cause of HCC worldwide, and the HBV regulatory protein X (HBx) acts as a positive regulator of STAT3 (Roca Suarez et al., 2018). Therefore, we also analyzed the anti-tumor effects of napabucasin on the HBV^+^ HepG2.2.15 cells. Consistent with the findings on Huh7 and Hepa1-6 cells, napabucasin significantly decreased the proliferation rates (Figure 5A), induced G2/M-phase arrest (Figure 5B), and increased apoptosis rates (Figures 5C,D) in the HepG2.2.15 cells in a time- and concentration-dependent manner. Notably, napabucasin could induce HepG2.2.15 cell apoptosis within 4 h (Supplementary Figure 3). Furthermore, napabucasin markedly reduced the colony forming capacity (Figure 5E) and blocked spherogenesis (Figure 5F) of HepG2.2.15 cells. The levels of Nanog, CD133, Klf4 and Sox-2 were increased in HepG2.2.15 spheroids (Figure 5G), which could be decreased by napabucasin treatment (Figure 5H). Interestingly, napabucasin also restricted HBV replication in the HepG2.2.15 cells by inhibiting HBx, HBV core protein (HBc), hepatitis B surface antigen (HBs) and HBV polymerase (HBp) (Figure 5I). Taken together, napabucasin can be considered for the treatment of HBV^+^ HCC.

### 3.5 Napabucasin Inhibits HCC Homografts *in vivo*

**FIGURE 6:**
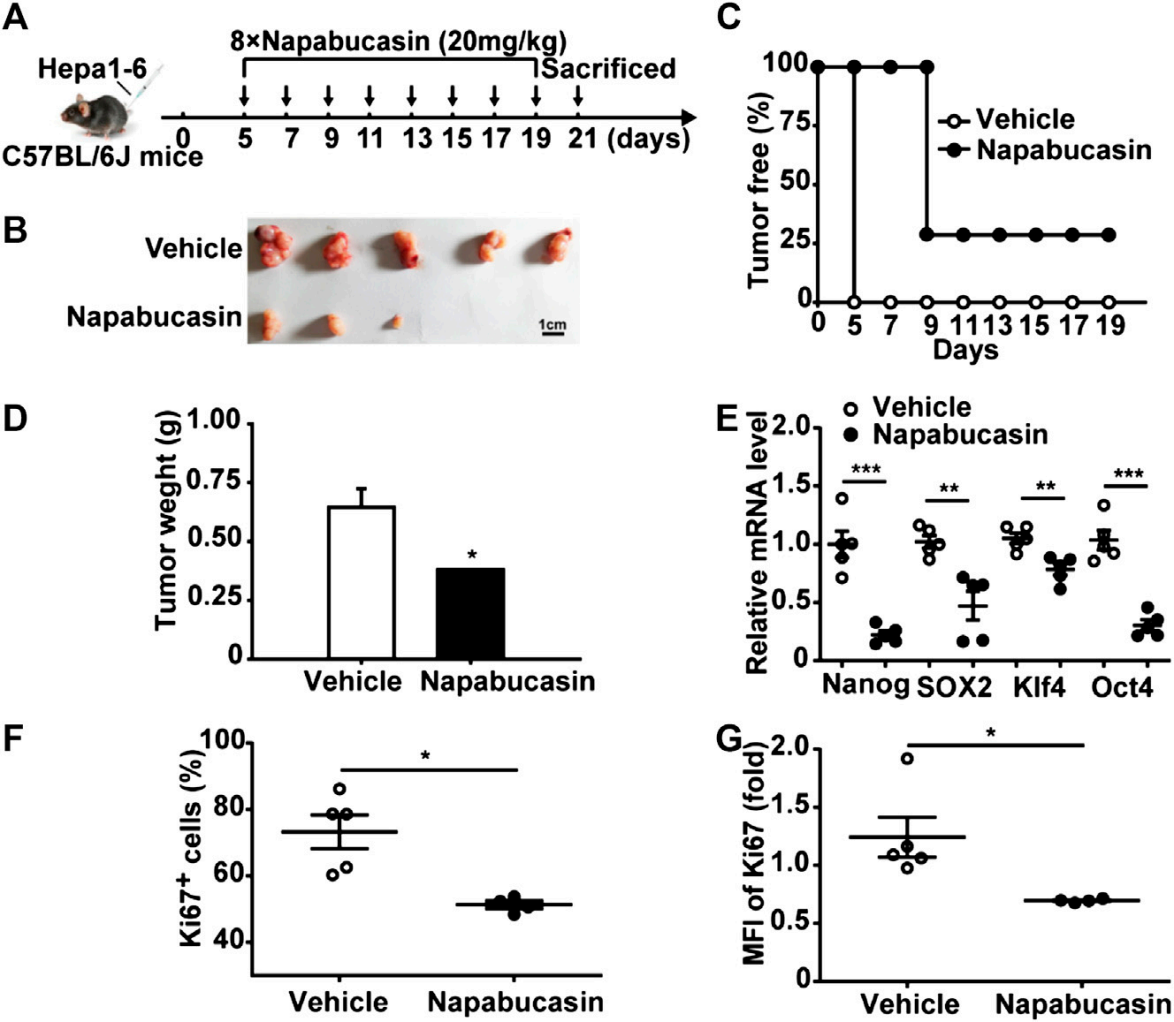
Napabucasin inhibits HCC homograft growth *in vivo*. **(A)** C57BL/6J mice were inoculated with 5 × 10^6^ Hepa1-6 cells in the left axilla, and intraperitoneally injected with napabucasin (20 mg/kg) or vehicle 5 days post-inoculation every 2 days for a total of eight injections. **(B)** Representative images of tumor tissues. **(C)** The percentage of tumor free mice. **(D)** Tumor weight of the suitably treated mice. **(E)** Stemness marker expression in the tumor cells. Data were normalized to β-actin. **(F)** Frequency of Ki67^+^ cells in the tumors. **(G)** Ki67 levels in tumor cells from mice treated as above. MFI, mean fluorescence index. Data represents the mean ± SD of n = 5 and were analyzed with one-way ANOVA (*P < 0.05, **P < 0.01 and ***P < 0.001).

**FIGURE 7:**
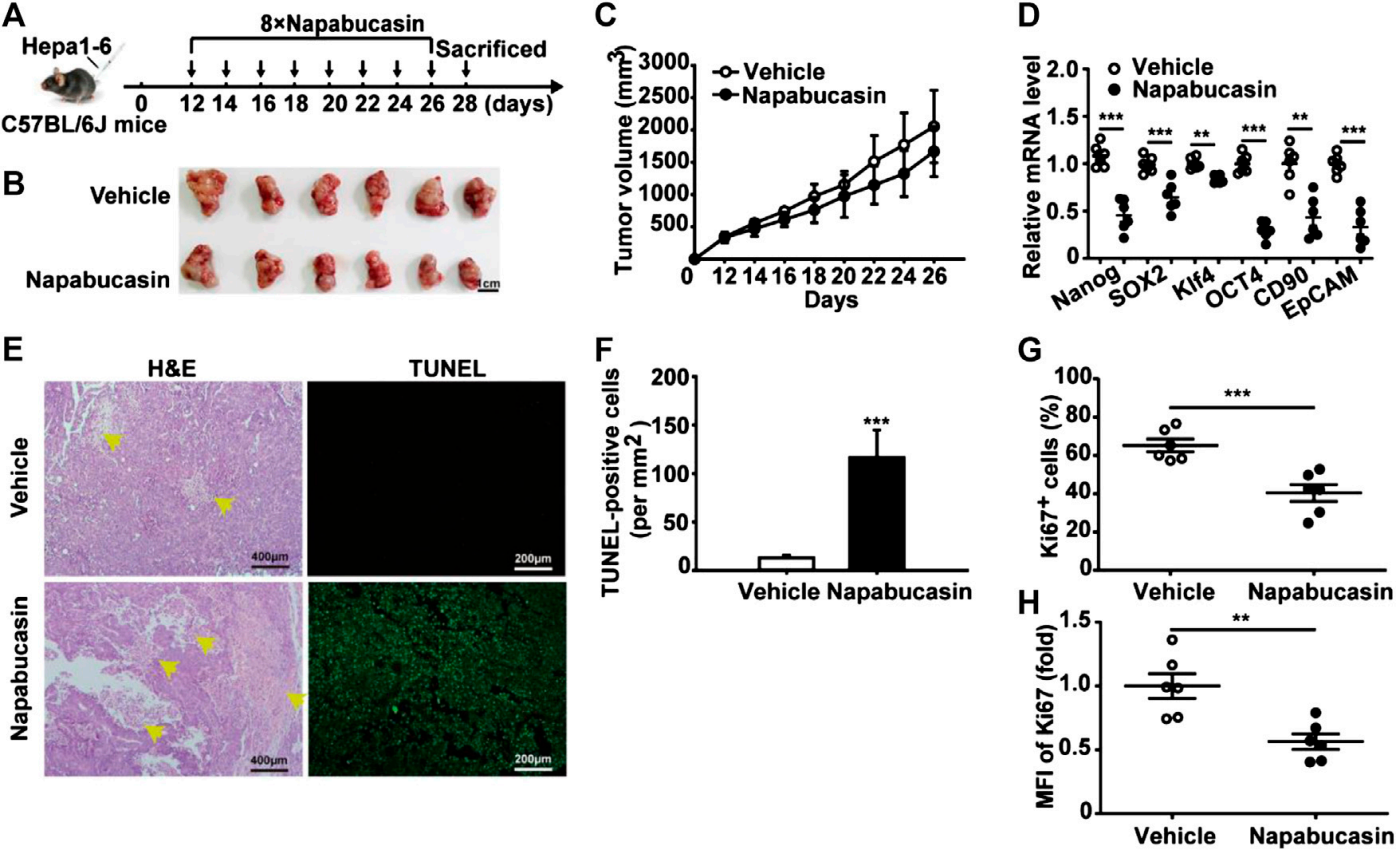
Napabucasin induces necrosis in Hepa1-6 homografts in advanced stages. **(A)** C57BL/6J mice were inoculated with 5 × 10^6^ Hepa1-6 cells in the left axilla, and intraperitoneally injected with napabucasin (20 mg/kg) or vehicle 12 days post-inoculation every 2 days for a total of eight injections. **(B)** Representative images of tumor tissues. **(C)** Tumor growth curves during the experimental period. **(D)** Stemness marker expression in tumor cells. Data were normalized to β-actin. **(E)** Representative images of H&E and TUNEL-stained tumor tissues. **(F)** Quantification of TUNEL-positive cells per mm^2^ in tumor tissues from suitably treated mice. **(G)** The frequency of Ki67^+^ cells in tumor tissues. **(H)** Ki67 levels in tumor cells. MFI, mean fluorescence index. Data represents the mean ± SD of n = 6 and were analyzed with ANOVA (**P < 0.01 and ***P < 0.001).

To evaluate the anti-HCC effect of napabucasin *in vivo*, Hepa1-6 homografts were established in mice that were subsequently treated with 20 mg/kg napabucasin or placebo (Figure 6A). Napabucasin treatment significantly reduced tumor volumes compared to that in the placebo group (Figure 6B), and the homografts regressed completely in two of the seven mice (Figure 6C). Furthermore, the final tumor weights in the vehicle and napabucasin groups were 0.645 ± 0.08 g and 0.38 ± 0.01 g respectively (Figure 6D). In line with the *in vitro* findings, napabucasin down-regulated Nanog, SOX2, Klf4 and Oct4 in the tumor cells (Figure 6E), and also decreased the proportion of Ki67^+^ tumor cells (Figures 6F,G). Since most HCC cases are detected in the advanced stages, we also analyzed the effect of napabucasin on the Hepa1-6 homografts 12 days post-inoculation (Figure 7A). As shown in Figures 7B and 7C, napabucasin did not significantly affect tumor growth compared to the placebo. However, napabucasin treatment increased the proportion of apoptotic tumor cells as well as the necrotic area in the tumor tissues (Figure 7D). Furthermore, napabucasin down-regulated Nanog, SOX2, Klf4, Oct4, CD90 and EpCAM, and decreased the percentage of Ki67^+^ cells in the tumors (Figures 7E–G). Taken together, napabucasin was effective against both early and advanced stage HCC homografts *in vivo*.

## 4 Discussion

Recent advances in surgical, systemic and locoregional therapies has improved the short-term survival of HCC patients. However, prognosis is still dismal due to high recurrence rates (Colagrande et al., 2016; Dimitroulis et al., 2017). CSCs are rare cells interspersed within the tumor bulk, and are responsible for chemo- and radio-resistance, and post-therapeutic relapse. In addition, long-term exposure to chemotherapeutic drugs or radiation often induces de-differentiation of mature cancer cells to stem-like phenotypes (Karakasiliotis and Mavromara, 2015; Chen et al., 2017a). Therefore, preventing CSCs formation is a viable strategy for preventing cancer relapse and metastasis.

The main cause of HCC-related mortality is the resistance to radiotherapies or chemotherapeutic drugs (Le Grazie et al., 2017). Furthermore, the advanced stage tumors are usually not resectable and therefore have limited treatment options. Sorafenib, currently the only targeted drug approved for systemic treatment of advanced HCC, confers a modest survival benefit (Dika and Abou-Alfa, 2017). Oxaliplatin is a platinum-based agent that blocks DNA replication and transcription, and has been approved for transcatheter arterial chemoembolization (TACE) along with epirubicin, oxaliplatin, mitomycin and lipiodol to treat HCC in China (Li et al., 2017). However, CSCs-induced resistance to oxaliplatin is a leading cause of combination treatment failure. Cryptotanshinone, a natural compound extracted from the root of *Salvia miltiorrhiza* Bunge, is a potential STAT3 inhibitor with anti-neoplastic effects (Chen et al., 2017b). It also induces apoptosis in the PGE2-treated HA22T HCC cells, and is therefore a novel drug for HCC treatment (Chang et al., 2018). Napabucasin effectively inhibits STAT3 and its downstream genes, including those regulating stemness (Li et al., 2015). It has been shown to suppress spherogenesis of or kill stemness-high cells isolated from head and neck, colon and pancreatic cancer cell lines (MacDonagh et al., 2018; Beyreis et al., 2019; Han et al., 2019). Furthermore, napabucasin also prevented metastasis in a spontaneous liver metastasis model of colorectal cancer, and suppressed relapse in a pancreatic cancer xenograft model (Li et al., 2015). We found napabucasin showed stronger cytotoxic effects on HCC cells compared to oxaliplatin and cryptotanshinone, and its IC_50_ was significantly lower.

CSCs drive cancer initiation, progression, metastasis, recurrence and drug resistance (Chen et al., 2013), and express Nanog, CD133, CD90, SOX2, EpCAM, CD44, Klf4 and Oct4, some of which may functionally support liver CSC phenotypes like invasiveness and chemoresistance (Yamashita and Wang, 2013). For instance, EpCAM^+^ HCC patients have lower overall and relapse free survival rates compared to the EpCAM^-^ HCC patients (Sung et al., 2016; Zhou et al., 2016). In addition, high levels of CD133 and CD44 are associated with unfavorable prognosis of HCC (Zhao et al., 2016). CD133^+^ Huh7 or SMMC-7721 cells show higher proliferative and tumorigenic capacity compared to the CD133^-^ counterparts (Yin et al., 2007). Since the CSCs directly accelerate cancer relapse and metastasis, it is necessary to identify and target stemness-related molecules for effective anti-cancer therapy (Baccelli and Trumpp, 2012).

STAT3 is constitutively active in CSCs independent of the upstream signaling regulators. In addition, CSCs or stemness-high cancer cells are extremely sensitive to the direct inhibition of STAT3 but insensitive to the inhibition of upstream factors like Janus kinases (Li et al., 2015). Napabucasin diminished the CSC-like traits of HCC cells, such as colony formation ability and spherogenesis, and killed the CSCs derived from Huh7 and Hepa1-6 spheres. Furthermore, napabucasin significantly downregulated the key stemness factors Nanog, SOX2, Oct4 and CD90 that are essential for maintaining pluripotency. Thus, napabucasin can inhibit HCC growth by restraining the CSCs.

Hepatitis B and hepatitis C infection strongly predispose patients to chronic liver diseases and HCC, and are the major etiological factors of HCC in Asia and Africa (McGlynn and London, 2011; Mittal and El-Serag, 2013). Liver tumor biopsies from HBV-infected patients show EpCAM overexpression, and increased percentage of Oct4^+^ and Nanog^+^ cells (Arzumanyan et al., 2011). Consistent with this, ectopic expression of the HBx protein in HepG2 cells significantly upregulated Nanog, Klf4, Oct4, EpCAM and *β*-catenin (Arzumanyan et al., 2011). In a previous study, we found that blocking STAT3 signaling inhibited HBV^+^ HCC cell growth *in vitro* by promoting apoptosis and cell cycle arrest. STAT3 silencing inhibited HBV replication, which decreased HBV-dependent activation of STAT3 through a negative feedback loop, and augmented the anti-HCC effects (Yang et al., 2016). Napabucasin effectively suppressed the viability, clonogenic ability and stemness of the HBV^+^ HepG2.2.15 cells, induced apoptosis and inhibited HBV replication, indicating the potential efficacy of napabucasin against HBV^+^ HCC.

The effects of napabucasin were also tested on early and advanced stage Hepa1-6 homografts *in vivo*. In the early stage model, napabucasin significantly inhibited tumor growth and decreased the percentage of proliferating cells, along with downregulating the stemness-related factors in the tumor tissues. However, napabucasin only marginally affected the growth of advanced tumor homografts despite inducing apoptosis and necrosis. It is possible that the effects of napabucasin in the advanced stages of HCC depend on its dose and bioavailability. Thus, dose-rate effects will have to be analyzed in order to improve the anti-tumor efficacy of napabucasin. Further, tumor tissues are composed of multiple cell types, including tumor cells, immune cells, endothelial cells, fibroblasts and so on, all of which are involved in the tumor progression (Fridman et al., 2012). For instance, a large number of regulatory T cells infiltrate in the tumor microenvironment, which is associated with the suppressive immune response to HCC cells (Togashi et al., 2019). Whether napabucasin can influence regulatory T cells or other cells in addition to HCC cells and then inhibit tumor growth by improving the tumor microenvironment need to be further explored.

**FIGURE 8:**
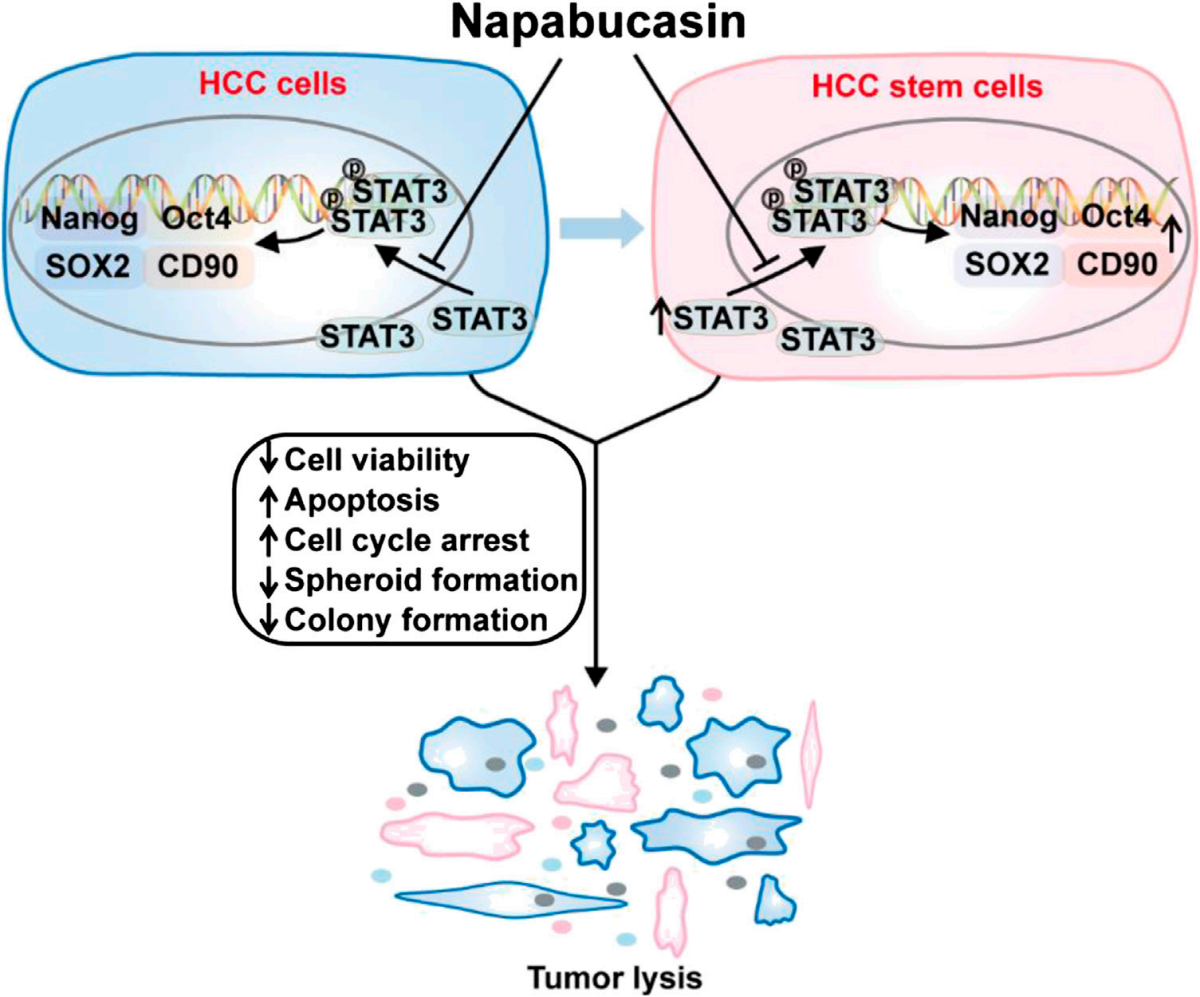
Napabucasin reduces cancer stem cell characteristics in hepatocellular carcinoma. Napabucasin significantly decreased the viability of HCC cells *in vitro* by inducing apoptosis and cell cycle arrest. In addition, it suppressed CSC-related gene expression and spheroid formation *in vitro*, indicating depletion of CSCs. Further, napabucasin also restricted HBV replication in HBV^+^ HCC cells.

In this study, we confirmed that napabucasin significantly decreased the viability of HCC cells *in vitro* by inducing apoptosis and cell cycle arrest. In addition, it suppressed CSC-related gene expression and spheroid formation *in vitro*, indicating depletion of CSCs. Interestingly, napabucasin also restricted HBV replication in HBV^+^ HCC cells. The anti-neoplastic effects of napabucasin was also evident in homograft tumor-bearing mouse models (Figure 8). Napabucasin is promising as a drug or an adjuvant to standard chemo/immune-therapeutics against HCC.

## Data Availability Statement

The original contributions presented in the study are included in the article/Supplementary Material, further inquiries can be directed to the corresponding author.

## Ethics Statement

The animal study was reviewed and approved by Ethical Committee of Shandong University.

## Author Contributions

YL: study conception, design and drafting of the manuscript. QH and HZ: lab work. QG: acquisition of data. JZ: study conception, design and critical revision.

## Funding

The work was supported by grants from National Natural Science Foundation of China (No. 81972694, No. 81972686) and the National Major Science & Technology Project for Control and Prevention of Major Infectious Diseases in China (No. 2018ZX10301401).

## Conflict of Interest

The authors declare that the research was conducted in the absence of any commercial or financial relationships that could be construed as a potential conflict of interest.
