# Supplementary material for: Napabucasin Reduces Cancer Stem Cell Characteristics in Hepatocellular Carcinoma
Source: Front Pharmacol. 2020 Dec 3;11:597520. doi: 10.3389/fphar.2020.597520 (PMC7744694; doi:10.3389/fphar.2020.597520)
Supplement: Supplementary file 1 [file datasheet1.pdf]

Certificate of Analysis
Cryptotanshinone

Research Area: JAK/STAT > STAT > Cryptotanshinone

Product Name:	Cryptotanshinone
Catalog Number:	S2285
Batch Number:	S228506

Physical and chemical properties

Molecular Formula:	$C_{19}H_{20}O_3$
Molecular Weight:	296.36
CAS No.:	35825-57-1
Stability:	3 years -20°C powder
	2 years -80°C in solvent
Molecular Structure:	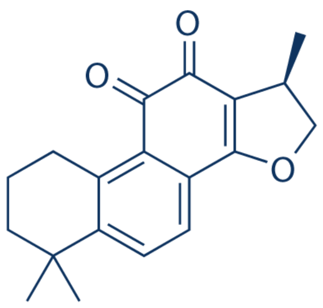

Analytical data

HPLC: 99.04% purity

NMR: Consistent with structure

Sample Name: F10563 S228506 Cryptotanshinone

===

Acq. Operator : SYSTEM Seq. Line : 11

Acq. Instrument : 1260lc Location : Vial 2

Inj Volume : 2.000 µl

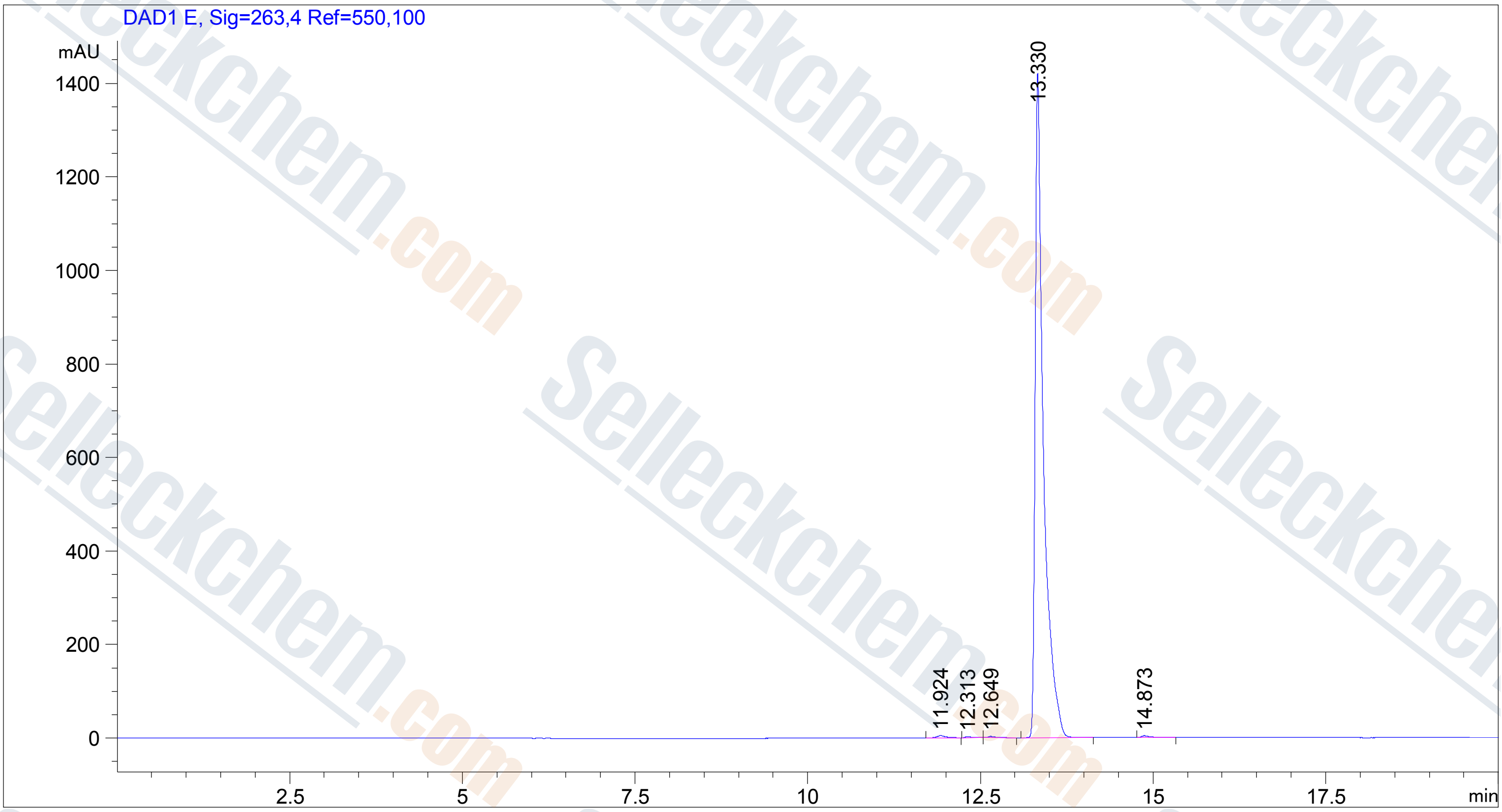

===

Area Percent Report

===

Sorted By : Signal

Multiplier : 1.0000

Dilution : 1.0000

Use Multiplier & Dilution Factor with ISTDs

Signal 1: DAD1 E, Sig=263,4 Ref=550,100

Peak #	RetTime [min]	Type	Width [min]	Area [mAU*s]	Height [mAU]	Area %
1	11.924	BB	0.1250	44.04285	4.87083	0.3677
2	12.313	BB	0.0960	15.11051	2.26016	0.1262
3	12.649	BB	0.1033	20.19618	2.73089	0.1686
4	13.330	BB	0.1150	1.18620e4	1420.04370	99.0421
5	14.873	BB	0.1225	35.37189	4.02773	0.2953

Totals : 1.19767e4 1433.93330

Sample Name: F10563 S228506 Cryptotanshinone

==
*** End of Report ***
