# Supplementary material for: Napabucasin Reduces Cancer Stem Cell Characteristics in Hepatocellular Carcinoma
Source: Front Pharmacol. 2020 Dec 3;11:597520. doi: 10.3389/fphar.2020.597520 (PMC7744694; doi:10.3389/fphar.2020.597520)
Supplement: Supplementary file 2 [file datasheet2.pdf]

Certificate of Analysis
Napabucasin(BBI608)

Research Area: JAK/STAT > STAT > Napabucasin(BBI608)

Product Name:	Napabucasin(BBI608)
Catalog Number:	S7977
Batch Number:	S797702

Physical and chemical properties

Molecular Formula:	$C_{14}H_{8}O_{4}$
Molecular Weight:	240.21
CAS No.:	83280-65-3
Stability:	3 years -20°C powder
	2 years -80°C in solvent

Molecular Structure:

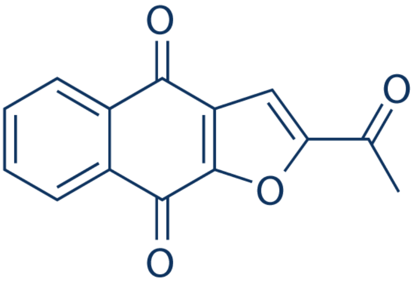

Analytical data

HPLC: 99.72% purity

NMR: Consistent with structure

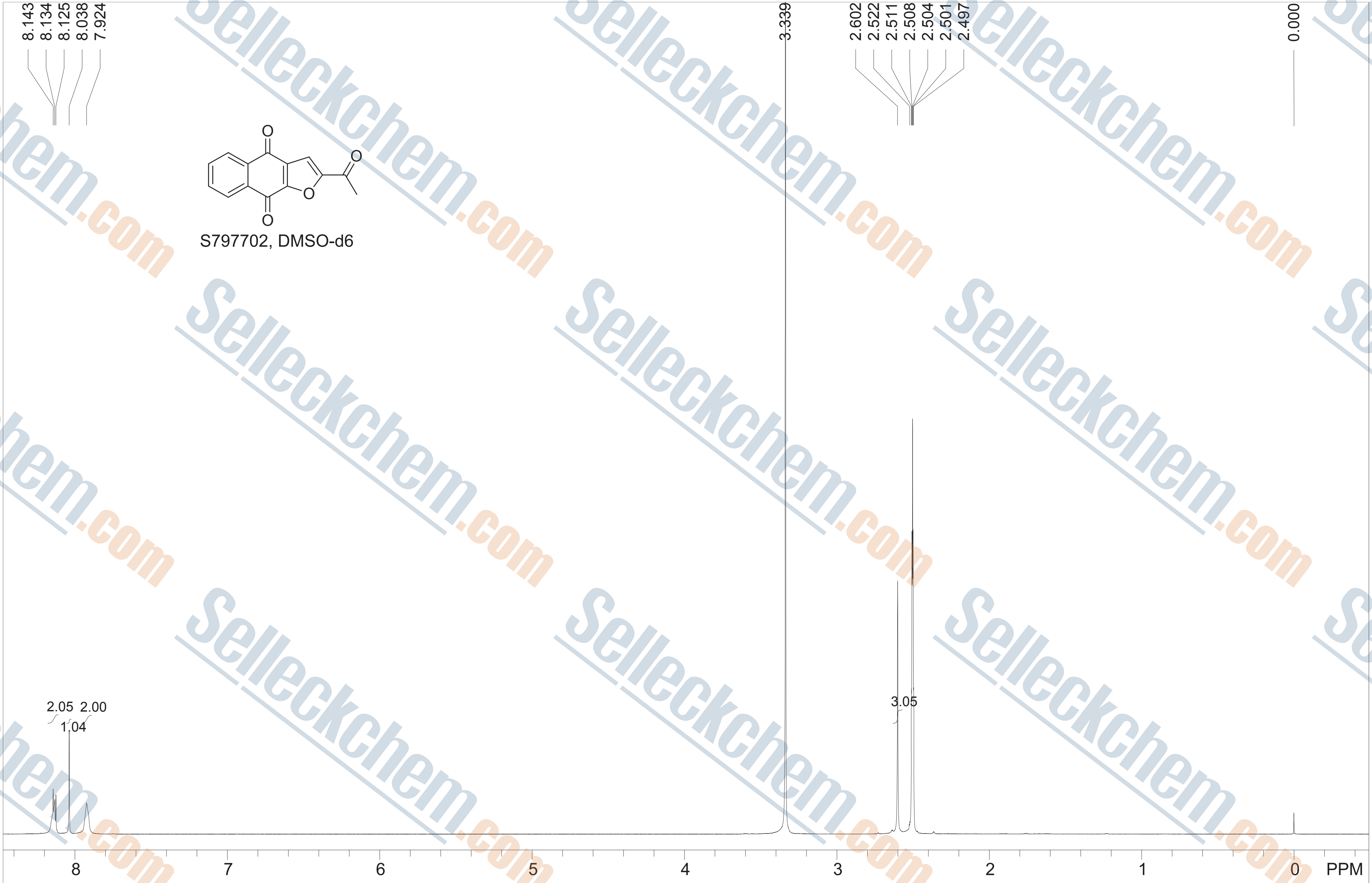

F1: 500.230	F2: 1.000	SW1: 10000		OF1: 3085.1		PTS1d: 65536	
EX: zg30		PW: 12.0 us	PD: 1.0 sec	NA: 8	LB: 0.0	Nuts - $S797702 Napabucasin(BBI608)_1.1	

Selleckchem.com

Sample Name: F8888 S797702 Napabucasin

===

Acq. Operator : SYSTEM Seq. Line : 1
Acq. Instrument : 1260lc Location : Vial 81

Inj Volume : 2.000 µl
Different Inj Volume from Sample Entry! Actual Inj Volume : 4.000 µl

Additional Info : Peak(s) manually integrated

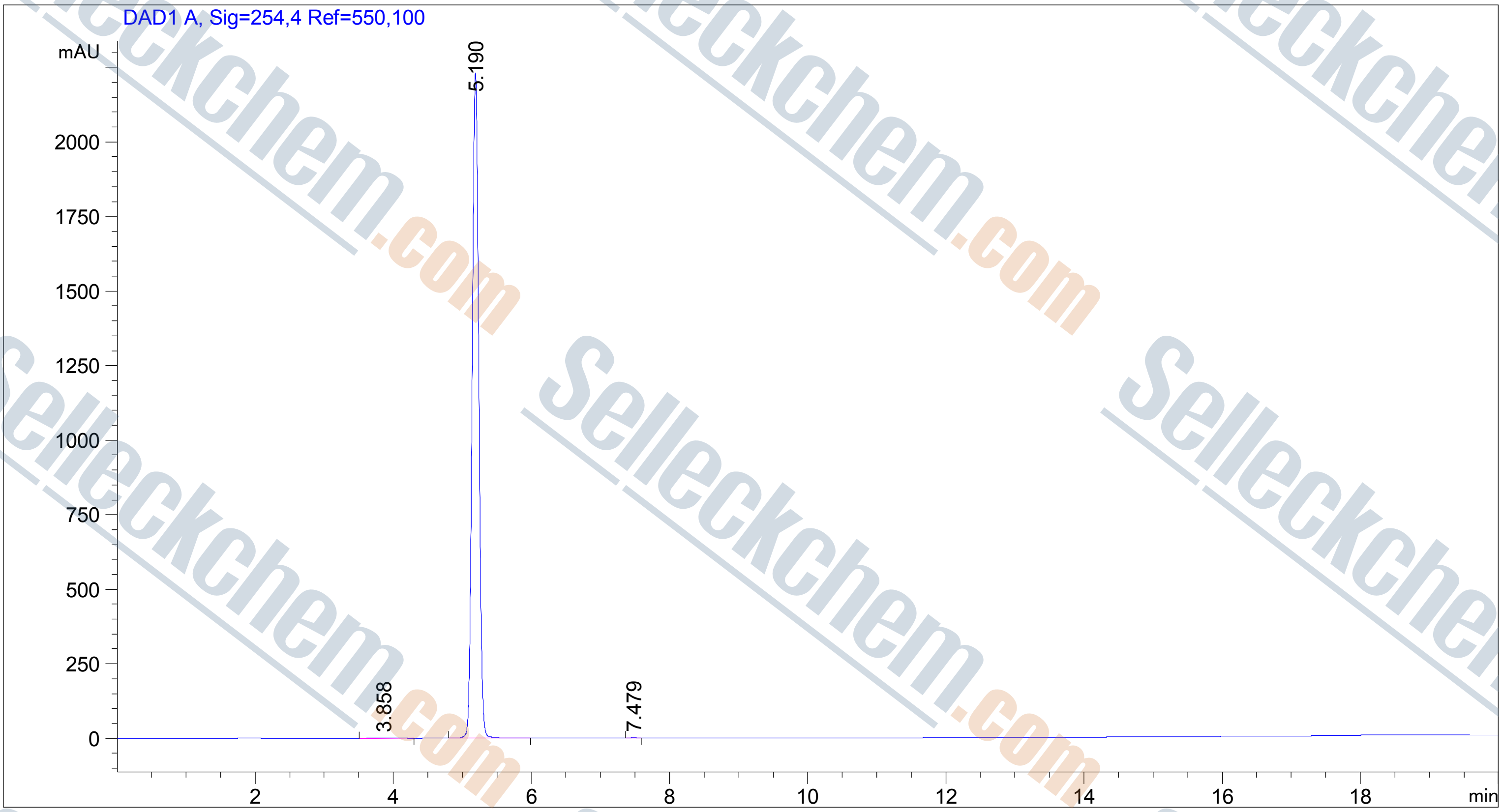

===

Area Percent Report

===

Sorted By : Signal
Multiplier : 1.0000
Dilution : 1.0000
Use Multiplier & Dilution Factor with ISTDs

Signal 1: DAD1 A, Sig=254,4 Ref=550,100

Peak #	RetTime [min]	Type	Width [min]	Area [mAU*s]	Height [mAU]	Area %
1	3.858	BB	0.2123	27.80548	1.61580	0.1874
2	5.190	BB	0.1045	1.47945e4	2228.43677	99.7275
3	7.479	BB	0.0612	12.62434	3.15366	0.0851

Totals : 1.48349e4 2233.20623

===
