# Supplementary material for: Napabucasin Reduces Cancer Stem Cell Characteristics in Hepatocellular Carcinoma
Source: Front Pharmacol. 2020 Dec 3;11:597520. doi: 10.3389/fphar.2020.597520 (PMC7744694; doi:10.3389/fphar.2020.597520)
Supplement: Supplementary file 3 [file datasheet3.pdf]

Certificate of Analysis
Oxaliplatin

Research Area: **DNA Damage/DNA Repair** > **DNA/RNA Synthesis** > **Oxaliplatin**

Product Name:	Oxaliplatin
Catalog Number:	S1224
Batch Number:	S122410

Physical and chemical properties

Molecular Formula:	$C_8H_{14}N_2O_4Pt$
Molecular Weight:	397.29
CAS No.:	61825-94-3
Stability:	3 years -20°C powder
	2 years -80°C in solvent
Molecular Structure:	

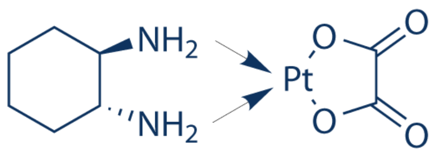

Analytical data

HPLC: 99.93% purity

NMR: Consistent with structure

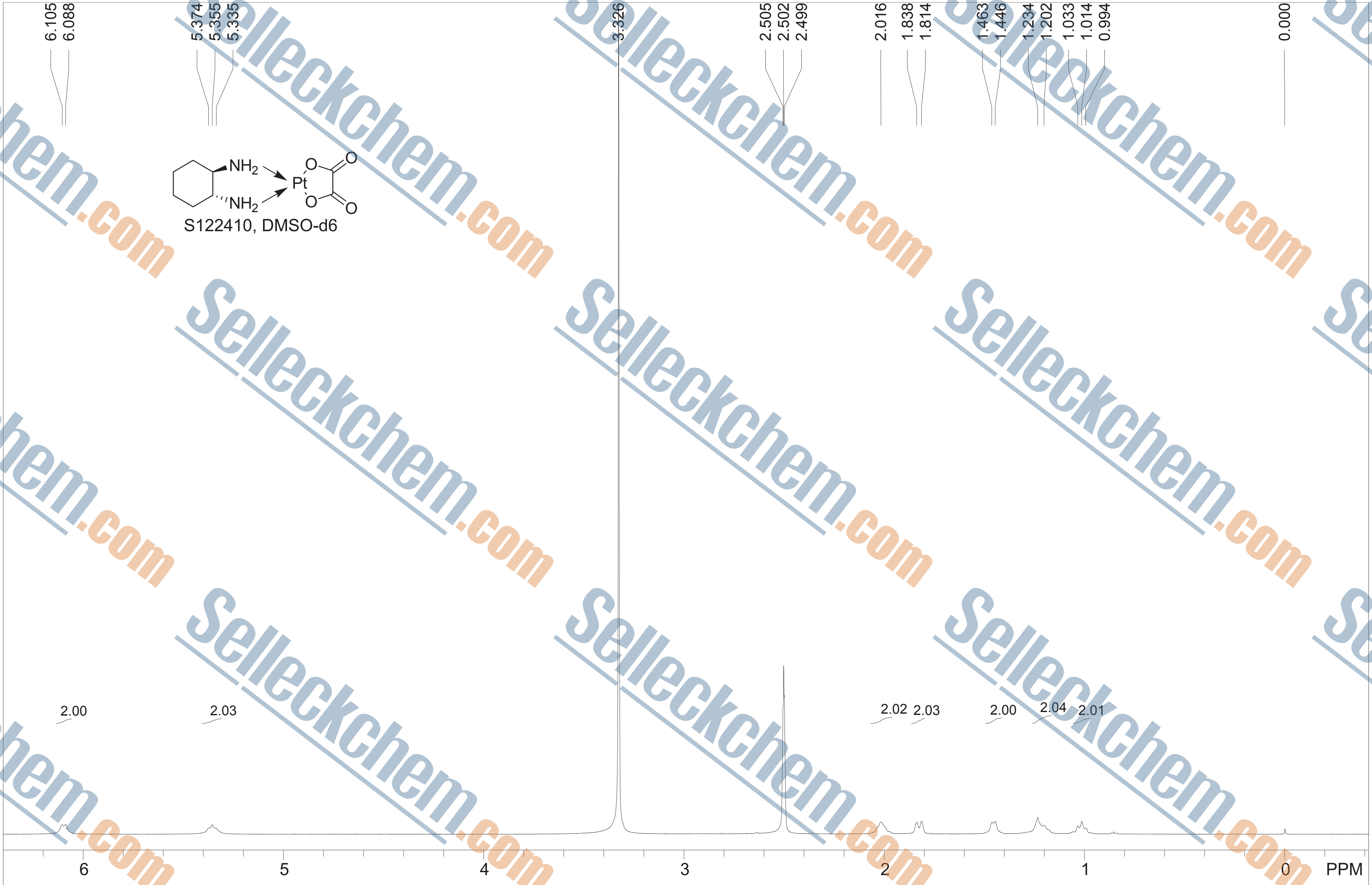

, , PROTON DMSO {D:\data} 1 18, , USER: nmrsu -- DATE: 14:18:31.945 +0800 nmrsu@CZC31189NY

F1: 500.230	F2: 1.000	SW1: 10000		OF1: 3072.3		PTS1d: 65536
EX: zg30		PW: 12.0 us	PD: 1.0 sec	NA: 8	LB: 0.3	Nuts - $S122410 OXALIPLATIN (oxaliplatin)_10.1

Selleckchem.com

Sample Name: F7339 S122410 OXALIPLATIN

===

Acq. Operator : SYSTEM Seq. Line : 1

Acq. Instrument : 1260lc Location : Vial 52

Inj Volume : 2.000 µl

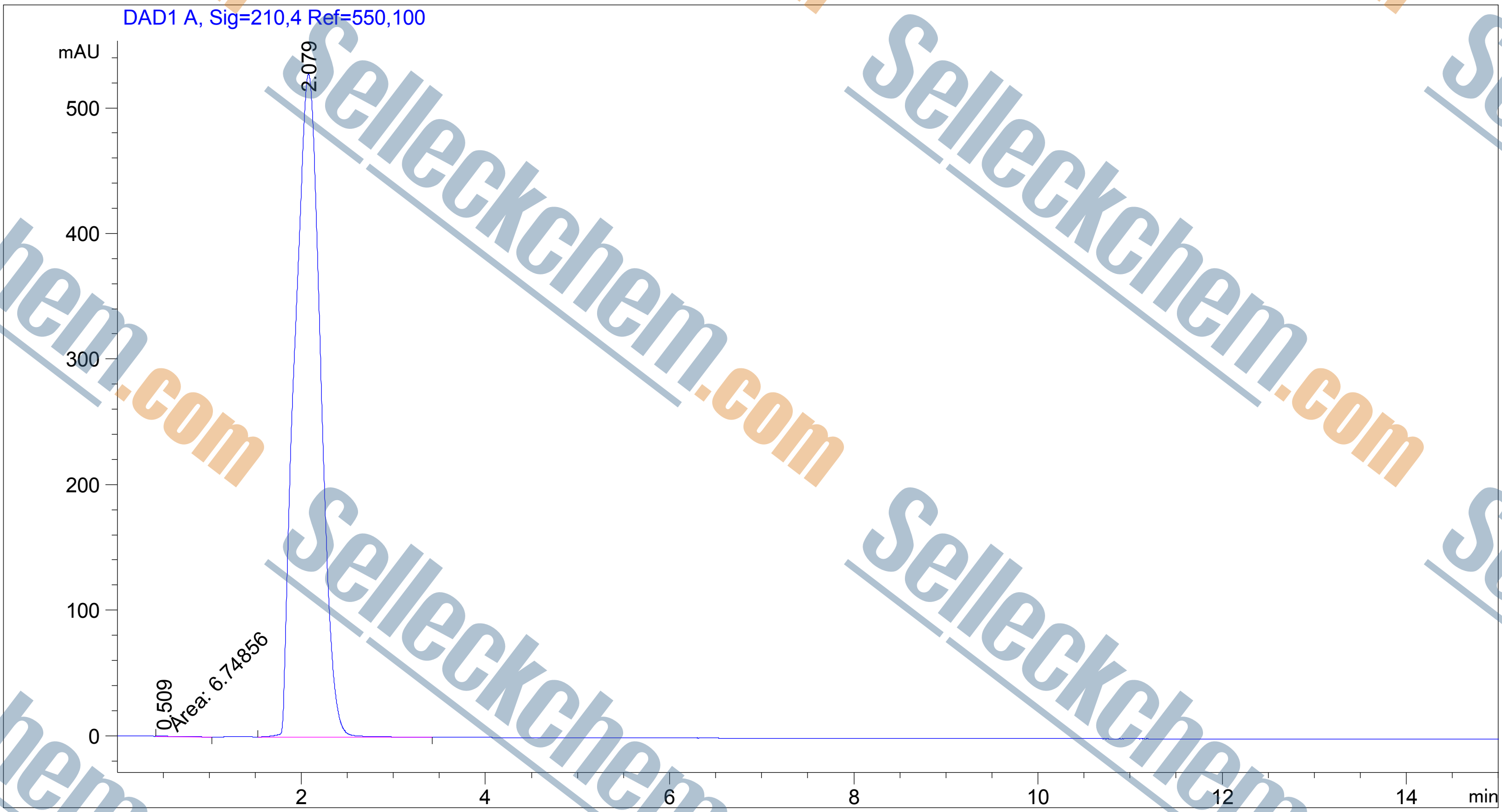

===

Area Percent Report

===

Sorted By : Signal

Multiplier : 1.0000

Dilution : 1.0000

Use Multiplier & Dilution Factor with ISTDs

Signal 1: DAD1 A, Sig=210,4 Ref=550,100

Peak #	RetTime [min]	Type	Width [min]	Area [mAU*s]	Height [mAU]	Area %
1	0.509	MM	0.3631	6.74856	3.09739e-1	0.0662
2	2.079	BB	0.2866	1.01914e4	528.17542	99.9338

Totals : 1.01982e4 528.48515

===

*** End of Report ***

Selleckchem.com
