# Supplementary material for: Napabucasin Reduces Cancer Stem Cell Characteristics in Hepatocellular Carcinoma
Source: Front Pharmacol. 2020 Dec 3;11:597520. doi: 10.3389/fphar.2020.597520 (PMC7744694; doi:10.3389/fphar.2020.597520)
Supplement: Supplementary file 4 [file datasheet4.docx]

**Napabucasin reduces cancer stem cell characteristics in hepatocellular carcinoma**

Ya Li^1^, Qiuju Han^1^, Huajun Zhao^1^, Quanjuan Guo^1^, Jian Zhang^1^

^1^Institute of Immunopharmaceutical Sciences, School of Pharmaceutical Sciences, Shandong University, Jinan, China

**Supplementary information:**


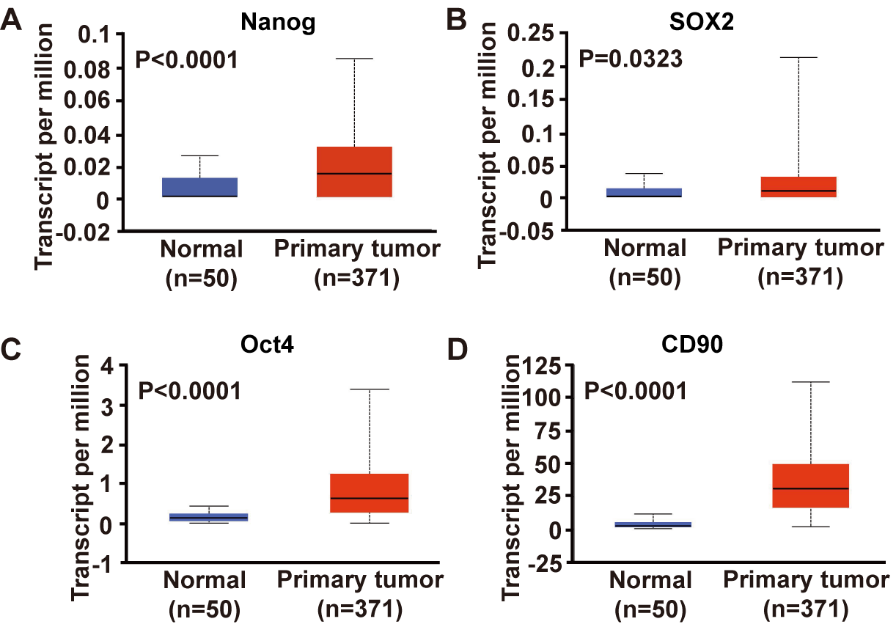


**Supplementary Figure 1** TCGA database analysis of Nanog (A), SOX2 (B), Oct4 (C) and CD90 (D) expression in HCC patients based on sample types through UALCAN database. The “primary tumors” are tumors from hepatocellular carcinoma patients, and “normal” sample represents normal liver tissues.

**
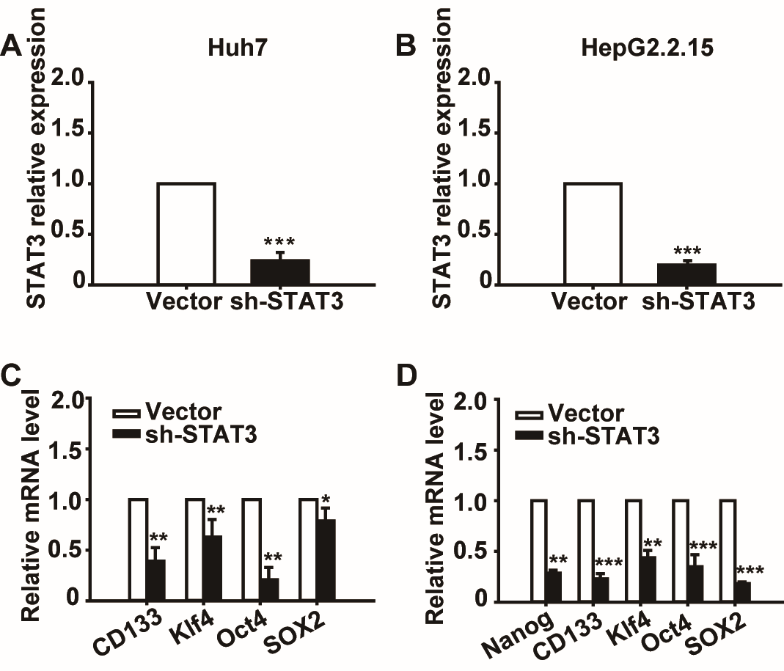
**

**Supplementary Figure 2 Inhibition of stemness markers by knockdown of STAT3 expression in HCC cells.** Lentivirus carrying STAT3-shRNA was used to interfere with the expression of STAT3 in Huh7 (A) and HepG2.2.15 (B) cells. The stemness marker expression in Huh7 (C) and HepG2.2.15 (D) cells. Data are shown as mean ± SD of three independent experiments (* P<0.05, ** P<0.01 and *** P<0.001).

**
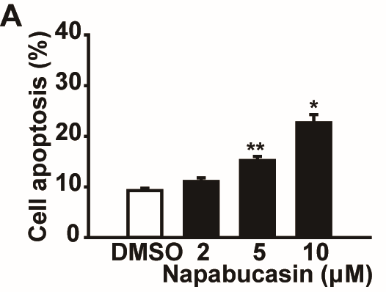
**

**Supplementary Figure 3** **Napabucasin induces HBV^+^ HCC cell apoptosis.** Percentage of apoptotic HepG2.215 cells following treatment with DMSO or napabucasin for 4h.

**Supplementary Table 1. Primer sequences for qRT-PCR.**

| Target | Species | Sequence(5′-3′) |
| --- | --- | --- |
| β-actin | human | Forward:CATGTACGTTGCTATCCAGGC |
|  |  | Reverse:CTCCTTAATGTCACGCACGAT |
| STAT3 | human | Forward:GAGGACTGAGCATCGAGCA |
|  |  | Reverse:CATGTGATCTGACACCCTGAA |
| Nanog | human | Forward:TTTGTGGGCCTGAAGAAAACT |
|  |  | Reverse:AGGGCTGTCCTGAATAAGCAG |
| SOX2 | human | Forward:GCCGAGTGGAAACTTTTGTCG |
|  |  | Reverse:GGCAGCGTGTACTTATCCTTCT |
| Klf4 | human | Forward:CGGACATCAACGACGTGAG |
|  |  | Reverse:GACGCCTTCAGCACGAACT |
| Oct4 | human | Forward:GTGTTCAGCCAAAAGACCATCT |
|  |  | Reverse:GGCCTGCATGAGGGTTTCT |
| CD133 | human | Forward:AGTCGGAAACTGGCAGATAGC |
|  |  | Reverse:GGTAGTGTTGTACTGGGCCAAT |
| β-actin | mouse | Forward:GGCCAACCGTGAAAAGATGA |
|  |  | Reverse:CACAGCCTGGATGGCTACGT |
| STAT3 | mouse | Forward:CAATACCATTGACCTGCCGAT |
|  |  | Reverse:GAGCGACTCAAACTGCCCT |
| Nanog | mouse | Forward:GAATCAGGGCTGCCTTGAAGAG |
|  |  | Reverse:CAGCAGATCACTCACATCGCCA |
| SOX2 | mouse | Forward:AACGGCAGCTACAGCATGATGC |
|  |  | Reverse:CGAGCTGGTCATGGAGTTGTAC |
| Klf4 | mouse | Forward:CTATGCAGGCTGTGGCAAAACC |
|  |  | Reverse:TTGCGGTAGTGCCTGGTCAGTT |
| Oct4 | mouse | Forward:CAGCAGATCACTCACATCGCCA |
|  |  | Reverse:GCCTCATACTCTTCTCGTTGGG |
| CD90 | mouse | Forward:TGCTCTCAGTCTTGCAGGTG |
|  |  | Reverse:TGGATGGAGTTATCCTTGGTGTT |
| EpCAM | mouse | Forward:GCGGCTCAGAGAGACTGTG |
|  |  | Reverse:CCAAGCATTTAGACGCCAGTTT |
| HBc |  | Forward:CTCTGTATCGGGAAGC |
|  |  | Reverse:TTAGGCCCATATTAGTG |
| HBx |  | Forward:CCGTCTGTGCCTTCTCATCTGC |
|  |  | Reverse:ACCAATTTATGCCTACAGCCTCC |
| HBs/p |  | Forward:ATCCTGCTGCTATGCCTCATCTT |
|  |  | Reverse:ACAGTGGGGGAAAGCCCTACGAA |
